# Case Report: Two-stage surgical management of spontaneous oesophageal rupture with extensive necrosis

**DOI:** 10.3389/fmed.2026.1902558

**Published:** 2026-09-09

**Authors:** Fu-Hui Chen, Bin Han, Yu-Cheng Wei, Yu-Hong Liu, Shuo Li, Gui-Song Song, Xiao-Tong Liu, Yang Li, Tian-Rui Fu

**Affiliations:** Department of Thoracic Surgery, The Affiliated Hospital of Qingdao University, Qingdao, China

**Keywords:** case report, gastric tube, oesophageal exclusion, oesophageal reconstruction, spontaneous oesophageal rupture

## Abstract

Spontaneous oesophageal rupture is a rare and life-threatening condition requiring urgent intervention. Management is particularly challenging when the rupture is accompanied by extensive transmural necrosis and severe thoracic contamination. We report a male patient in his late 30s who underwent emergency treatment approximately 11 h after symptom onset and was found to have an 8-cm full-thickness oesophageal tear with extensive transmural necrosis. Given the severe mediastinal and thoracic contamination and marked friability of the oesophageal wall, primary repair was considered unsuitable. A staged surgical strategy was therefore adopted. Stage I involved resection/exclusion of the damaged thoracic oesophagus with cervical oesophageal exteriorisation, gastrostomy for gastric drainage and decompression, and jejunostomy for enteral nutritional support. Stage II, performed 4 months later, consisted of gastrointestinal reconstruction using a retrosternal gastric conduit. During the 18-month follow-up, the patient resumed unrestricted oral intake and showed favourable nutritional and functional recovery. This case suggests that a staged surgical strategy may represent a feasible treatment option in carefully selected patients with severe Boerhaave syndrome when secure primary repair is precluded by extensive oesophageal necrosis and severe thoracic contamination.

## 1 Introduction

Spontaneous oesophageal rupture is a rare but highly fatal oesophageal emergency (1). It predominantly affects young and middle-aged men, with a male-to-female ratio of approximately 5:1. The exact pathogenesis remains unclear but is closely related to the anatomical characteristics of the oesophagus and a sudden increase in intraluminal pressure (2).

Although various surgical interventions for spontaneous oesophageal rupture have been described, the optimal management of patients with extensive transmural necrosis, severe tissue friability, and gross thoracic contamination remains uncertain (3, 4). Primary repair or endoscopic treatment may be difficult when the oesophageal wall is extensively non-viable and unable to support secure closure. This report describes a staged surgical strategy used in a patient with extensive oesophageal necrosis and severe bilateral thoracic contamination in whom primary repair was considered technically unsuitable.

## 2 Case presentation

### 2.1 Patient information and initial assessment

A male patient in his late 30s was admitted to the emergency department on May 28, 2019, with sudden chest tightness and chest pain for more than 10 h. The patient had experienced severe nausea and vomiting after eating on May 27, 2019. He had a history of gastric bleeding treated with interventional embolisation of the left gastric artery in 2015.

On physical examination, the patient appeared lethargic and pale, with a temperature of 37 °C, heart rate of 91 beats/min, and blood pressure of 113/80 mmHg. Subcutaneous emphysema was palpable at the root of the neck. Breath sounds were diminished in both lower lungs with moist rales.

### 2.2 Diagnostic assessment

**Figure 1:**
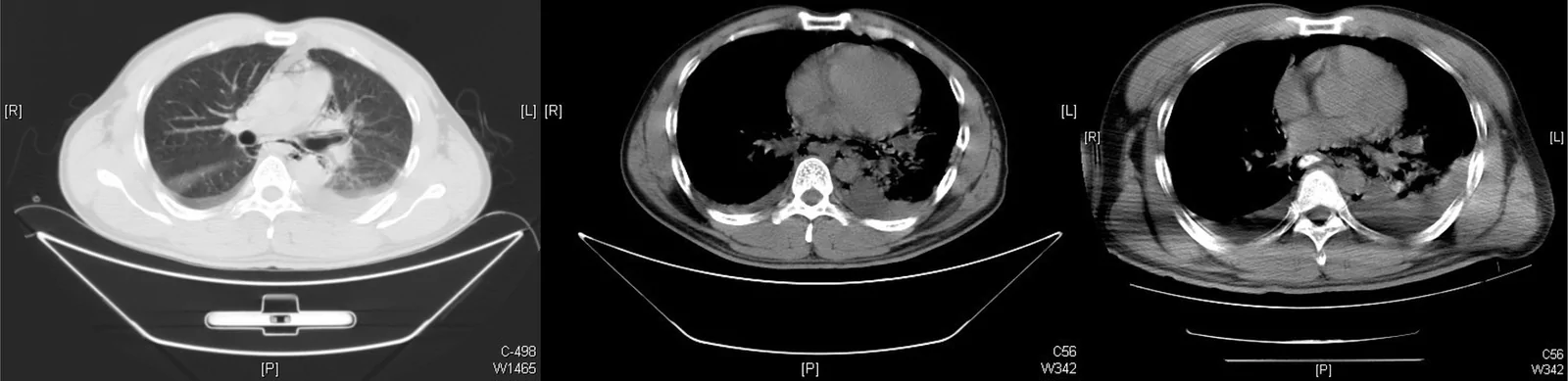
Chest CT on admission showing extensive pneumomediastinum and pleural effusion.

Emergency chest CT on admission showed cervical and mediastinal emphysema, bilateral pleural effusion with adjacent lung atelectasis, and inflammatory changes in the bilateral lower lobes (Figure 1). Abdominal CT was unremarkable. Laboratory investigations upon admission revealed significant systemic inflammation: a white blood cell count of 10.50 × 10⁹/L (normal range: 3.5–9.5 × 10⁹/L), a neutrophil percentage of 85% (normal: 40%–75%), C-reactive protein (CRP) of 113 mg/L (normal: < 8 mg/L), and procalcitonin (PCT) of 8.69 ng/mL (normal: < 0.05 ng/mL).

After multidisciplinary consultation, spontaneous oesophageal rupture was highly suspected. Repeat chest CT after transfer to the thoracic department showed markedly increased cervical and mediastinal emphysema, increased bilateral pleural effusion, and worsened atelectasis (Figure 1). Combining the history, clinical manifestations, and imaging features, spontaneous oesophageal rupture was confirmed.

### 2.3 Therapeutic interventions

Emergency surgery was performed on May 28, 2019. After induction of general anaesthesia, upper gastrointestinal endoscopy revealed a large rupture along the left side of the thoracic oesophagus, with food debris and necrotic tissue within the oesophageal lumen. Endoscopic repair and stent placement could not be performed, and surgical treatment was therefore undertaken.

Stage I Operation: The patient was initially placed in the right lateral decubitus position, and thoracoscopic exploration of the left pleural cavity was performed. A large amount of thick purulent fluid, extensive fibrinopurulent deposits, and dense pleural adhesions were identified, which markedly restricted the operative space. A thoracoscopic-assisted left anterolateral thoracotomy was therefore used to provide adequate exposure for thorough debridement and safe mobilisation of the thoracic oesophagus. An approximately 8-cm full-thickness oesophageal tear with extensive ischaemic necrosis was identified. The normal muscular appearance of the oesophageal wall had disappeared, and both the mucosal and muscular layers showed diffuse dusky-purple ischaemic discoloration. The tissue fragmented readily when grasped, and no active capillary bleeding was observed after debridement. The remaining oesophageal wall was unable to hold sutures reliably and was therefore considered unsuitable for reinforced primary repair. After thorough debridement, the pleural cavity was irrigated with dilute povidone-iodine. The thoracic oesophagus was mobilised and transected distally at the cardia using a linear cutting stapler, and the distal stump was reinforced with continuous sutures. The patient was then repositioned in the left lateral decubitus position. Thoracoscopic exploration of the right pleural cavity revealed similarly severe contamination, and a thoracoscopic-assisted right anterolateral thoracotomy was performed for thorough debridement and irrigation. The oesophagus was mobilised superiorly and transected approximately 2 cm above the injured segment, allowing removal of the damaged oesophageal segment. After completion of the bilateral thoracic procedures, the patient was returned to the supine position. A midline laparotomy was performed. A gastrostomy was created for gastric drainage and decompression, whereas a feeding jejunostomy was established to provide long-term enteral nutritional support. A left cervical incision was then made, the residual upper thoracic oesophagus was removed, and the cervical oesophagus was exteriorised by suturing the oesophageal mucosa intermittently to the surrounding skin.

Postoperative Management: Management included intensive anti-infective therapy, adequate drainage, and enteral nutritional support through the jejunostomy. After 4 months, the infection was controlled, biochemical indices normalised, weight increased by 1.5 kg, and serum albumin reached 40 g/L.

**Figure 2:**
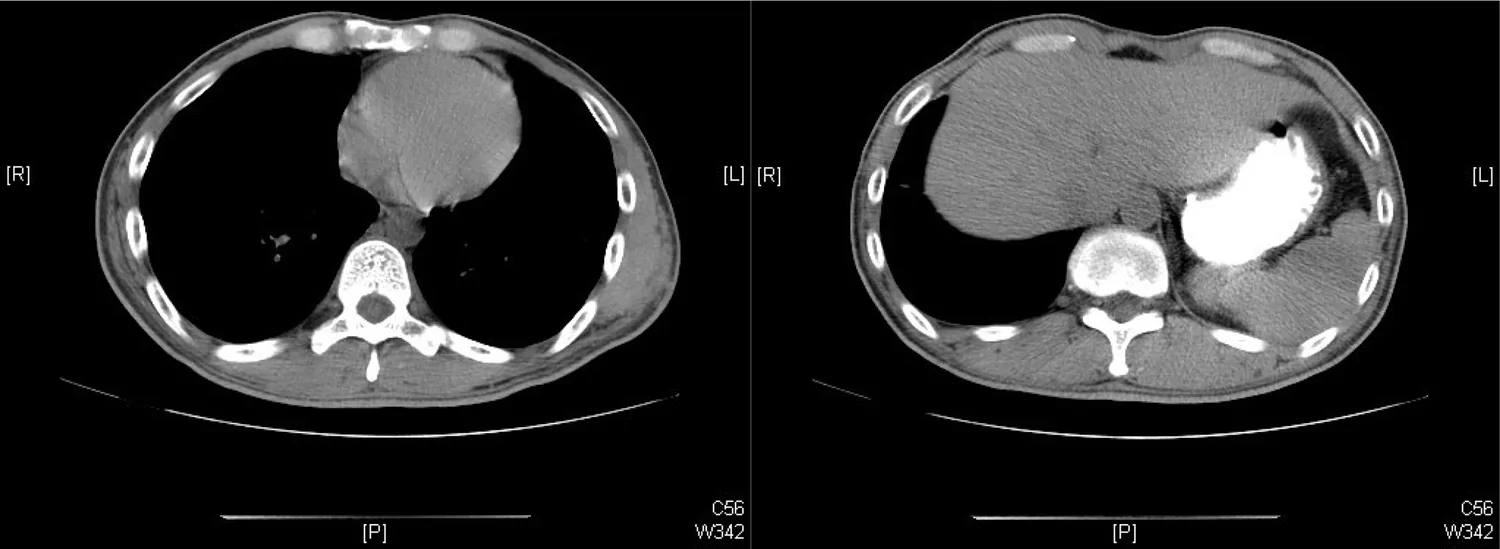
Preoperative chest CT prior to Stage II surgery showing resolution of pleural effusion and pneumomediastinum, disappearance of the oesophageal shadow, and satisfactory gastric distension.

Stage II Operation: Four months after the first-stage operation, follow-up CT demonstrated resolution of pneumomediastinum, pleural effusion, and thoracic infection (Figure 2), and gastrointestinal reconstruction was performed. The patient was placed in the supine position. The previous gastrostomy was closed. The jejunostomy tube was retained to provide postoperative enteral nutritional support and was subsequently removed after stable oral intake had been established. The right gastroepiploic vascular arcade was preserved as the principal blood supply to the gastric conduit. The stomach was mobilised along the greater curvature, and the lesser curvature was resected to create a narrow gastric tube. Because inflammatory adhesions had developed in the original oesophageal bed after the first-stage operation, a retrosternal route was selected. The gastric conduit was then brought to the neck through the retrosternal route and a tension-free side-to-side anastomosis was performed with the cervical oesophagus.

### 2.4 Outcomes and follow-up

**Figure 3:**
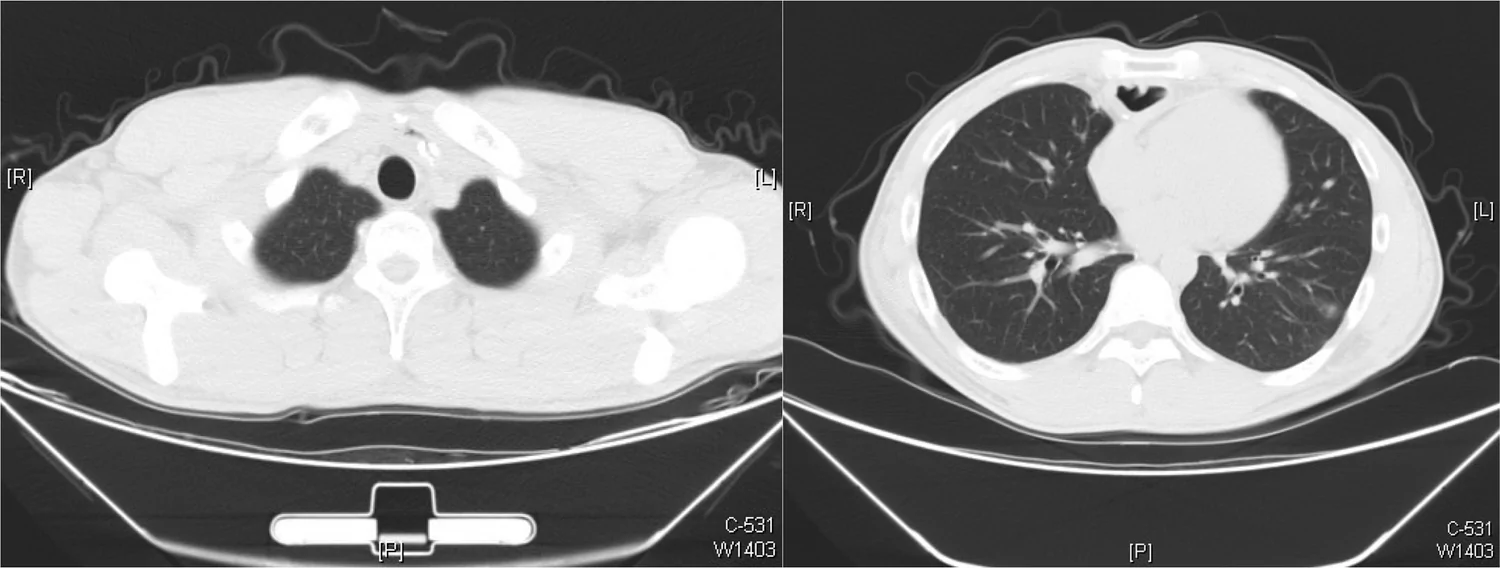
Follow-up chest CT after Stage II surgery showing complete resolution of pleural effusion and pneumomediastinum, well-expanded lungs, and visualisation of the retrosternal tubular gastric conduit.

**Figure 4:**
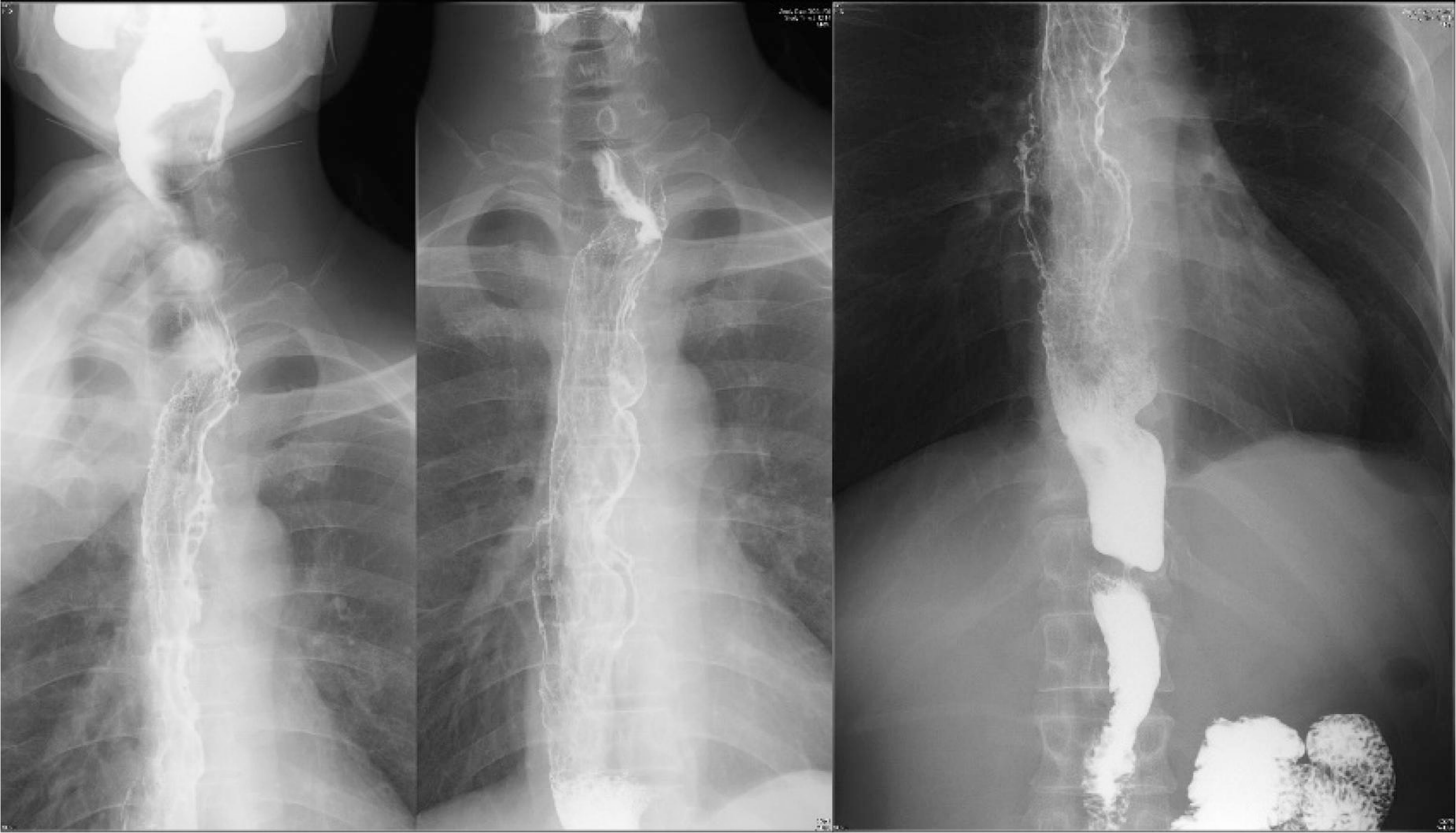
Follow-up upper gastrointestinal series after Stage II surgery demonstrating a well-shaped tubular stomach with smooth contrast transit and no evidence of contrast extravasation.

The postoperative course after the second-stage operation was uneventful. Serum albumin increased from 32 g/L before the first-stage operation to 40 g/L before the second-stage operation and reached 44 g/L at the 18-month follow-up. The patient gained a total of 7.2 kg after the two-stage treatment. Oral intake was resumed more than 20 days after the second-stage operation, and the patient was subsequently discharged in good condition (Figure 3). During the 18-month follow-up, the patient had no grade 2 or greater dysphagia according to the Ogilvie classification, no obvious reflux, and no nocturnal aspiration. Upper gastrointestinal contrast examination and cervical CT demonstrated a patent gastric conduit without contrast leakage or anastomotic stenosis (Figure 4). No gastric conduit necrosis, fistula, or recurrent mediastinal infection was observed during follow-up. The patient ultimately resumed unrestricted oral intake and returned to his pre-illness body weight. At outpatient follow-up, the patient reported that he was satisfied with the recovery of his digestive function. The EORTC QLQ-OG25 questionnaire was used during follow-up, with favourable findings in the digestive-related dimensions.

This case report was prepared in accordance with the Case Report (CARE) guidelines. Written informed consent was obtained from the patient for treatment. We have deidentified all patient details.

Clinical timeline: The patient presented acutely with spontaneous oesophageal rupture and underwent emergency Stage I oesophageal resection/exclusion. After 4 months of recovery, with control of thoracic infection and improvement in general and nutritional status, elective Stage II gastrointestinal reconstruction was performed. At 18-month follow-up, the patient had resumed unrestricted oral intake and showed favourable clinical recovery.

## 3 Discussion

Spontaneous oesophageal rupture is a rare but highly lethal disease, predominantly affecting young and middle-aged men. The pathogenesis is believed to be closely related to the anatomical features of the oesophagus and a sudden rise in intraluminal pressure. In this case, the patient had a history of severe vomiting after eating prior to onset, suggesting that the rupture was likely related to the sudden increase in intraluminal oesophageal pressure caused by vomiting.

Spontaneous oesophageal rupture often has an insidious onset and lacks specific clinical manifestations. It commonly presents with chest pain, chest tightness, dyspnoea, and subcutaneous emphysema. In severe cases, it can rapidly progress to septic or hypovolemic shock and must be differentiated from acute abdomen and cardiovascular diseases (2). Imaging examinations play a crucial role in diagnosis. Chest CT typically shows mediastinal emphysema and hydropneumothorax. Oral methylene blue or iodinated contrast oesophagography can clearly identify the location of the rupture. Gastroscopy can directly visualise the site and extent of the injury; however, its use remains controversial due to the potential risk of exacerbating the damage. In this patient, gastroscopy was performed under general anaesthesia without related complications and was of great significance in clarifying the location and extent of the oesophageal rupture as well as guiding the selection of the surgical approach.

Currently, there is no unified standard for the treatment of spontaneous oesophageal rupture, which mainly includes two major categories: nonoperative and surgical treatment. Nonoperative management is suitable for patients who cannot tolerate surgery or have relatively mild disease. The main measures include adequate anti-infective therapy, effective drainage, gastrointestinal decompression, and enteral nutritional support; however, the mortality rate remains high (5, 6). Therefore, most current studies tend to favour a comprehensive treatment strategy centred on surgical intervention (7, 8). Surgical options include endoscopic or interventional stent placement, direct surgical repair, one-stage gastrointestinal reconstruction, and stage I oesophageal exclusion combined with stage II gastrointestinal reconstruction (9–11).

Stent placement is appropriate for patients with small ruptures, mild infection, and short symptom duration. It can achieve infection control by sealing the rupture in combination with thoracic drainage and anti-infective therapy (12–15). Direct surgical repair is also suitable for patients with mild degrees of injury (16–18). For patients with larger ruptures but controllable infection and short duration of symptoms, one-stage gastrointestinal reconstruction may be considered (19, 20). However, for patients with extensive oesophageal necrosis, severe tissue friability, and severe thoracic contamination that preclude secure primary repair, staged oesophageal exclusion followed by delayed gastrointestinal reconstruction may be considered.

The choice of surgical strategy in this case was determined primarily by the severity of the local oesophageal injury rather than by the interval from symptom onset to surgery. The patient had an approximately 8-cm full-thickness tear associated with extensive transmural necrosis, marked tissue friability, and severe bilateral thoracic contamination. Intraoperatively, the affected oesophageal wall showed poor viability and was unable to retain sutures reliably. Under these circumstances, reinforced primary repair was considered technically unsuitable. The extensive length of the tear and necrosis of the involved oesophageal segment also made effective sealing with an oesophageal stent difficult. In addition, both pleural cavities contained large amounts of purulent material and food debris. Drainage alone would therefore not have eliminated the persistent source of contamination from the disrupted oesophagus. The damaged thoracic oesophageal segment was consequently resected during the first-stage operation, and the residual upper thoracic oesophagus was subsequently resected through the cervical approach before cervical oesophageal exteriorisation, thereby avoiding a retained closed thoracic oesophageal segment and the associated risks of mucocele formation and recurrent suppurative infection (21). Immediate gastrointestinal reconstruction was deferred because of the severe bilateral thoracic contamination. The first-stage procedure therefore focused on removal of the damaged oesophageal segment, control of thoracic contamination, adequate drainage, and nutritional support. Definitive gastrointestinal reconstruction was performed 4 months later, after the infection had resolved and the patient's general and nutritional condition had improved, with serum albumin increasing to 40 g/L.

Despite the successful outcome, this report describes only a single retrospective case involving a highly selected patient with extensive full-thickness oesophageal necrosis and severe bilateral pleural contamination. Therefore, the findings cannot be generalized to all patients with spontaneous oesophageal rupture or establish this staged strategy as a standard surgical approach for all severe cases. Further studies with larger cohorts and longer-term functional assessments of the retrosternal gastric conduit are required to further evaluate the broader applicability of this strategy.

## 4 Conclusion

In this carefully selected patient with spontaneous oesophageal rupture accompanied by extensive transmural necrosis, severe tissue friability, and severe thoracic contamination that precluded secure primary repair, a staged strategy consisting of initial oesophageal resection/exclusion followed by delayed retrosternal gastrointestinal reconstruction represented a feasible alternative to high-risk primary repair. This case may provide a clinical reference for the management of selected patients with severe Boerhaave syndrome.

## Data Availability

The original contributions presented in the study are included in the article/Supplementary Material, further inquiries can be directed to the corresponding authors.
